# Interfacial Rheological Investigation of Modified Silica Nanoparticles with Different Alkyl Chain Lengths at the n-Octane/Water Interface

**DOI:** 10.3390/molecules29163749

**Published:** 2024-08-07

**Authors:** Long Xu, Shijie Wen, Qiuyu Xie, Fangning Fan, Qiang Wang, Xuehao Zhang, Kaihe Lv, Han Jia, Hai Sun

**Affiliations:** 1 Shandong Key Laboratory of Oilfield Chemistry, School of Petroleum Engineering, China University of Petroleum (East China), Qingdao 266580, China; xulong@upc.edu.cn (L.X.);; 2 Key Laboratory of Unconventional Oil & Gas Development, China University of Petroleum (East China), Ministry of Education, Qingdao 266580, China

**Keywords:** modified silica nanoparticles, alkyl chain length, dilational rheology, adsorption and desorption, surface pressure

## Abstract

The interfacial dilational rheology of silica nanoparticles (NPs) directly reflects the relationship between surface structure and interfacial behaviors in NPs, which has attracted significant attention in various industrial fields. In this work, modified silica nanoparticles (MNPs) with various alkyl chain lengths were synthesized and systematically characterized using Fourier transform infrared spectra, Zeta potential, and water contact angle measurements. It was found that the MNPs were successfully fabricated with similar degrees of modification. Subsequently, the interfacial behaviors of the MNPs in an n-octane/water system were investigated through interfacial dilational rheological experiments. The length of the modified alkyl chain dominated the hydrophilic–lipophile balance and the interfacial activity of the MNPs, evaluated by the equilibrium interfacial tension (IFT) variation and dilational elasticity modulus. In the large amplitude compression experiment, the balance between the electrostatic repulsion and interfacial activity in the MNPs was responsible for their ordered interfacial arrangement. The MNPs with the hexyl alkyl chain (M6C) presented the optimal amphipathy and could partly overcome the repulsion, causing a dramatic change in surface pressure. This was further confirmed by the variations in IFT and dilational elasticity during the compression path. The study provides novel insights into the interfacial rheology and interactions of functionally modified NPs.

## 1. Introduction

In the past few decades, NPs with a large specific surface area, remarkable adsorption capacity, and high chemical activity have been widely applied in the field of medicine, biology, materials, energy [1,2,3,4], and especially enhancing oil recovery (EOR) in the petroleum industry [5,6]. Some studies have demonstrated the additional NPs could largely improve the stability of emulsions and foams, which can be attributed to the enhanced interfacial rheological properties of the systems [7,8,9]. The interfacial rheological property reflects the diffusion exchange of NPs between the bulk phase and interface and the intensity of the interfacial film, which is employed to investigate the interfacial behaviors of the NPs [10,11,12].

According to different forms of deformation, interfacial rheology can be divided into shear and dilational rheology [13,14]. Researchers pay more attention to interfacial dilational viscoelasticity because it determines the stability of emulsions and foams [15,16,17,18]. Nikolay A. et al. investigated the dynamic properties of layers of cupin-1.1 aggregates at the air/water interface and demonstrated that there were two maxima in the surface elasticity of these layers [19]. The original hydrophilic silica nanoparticles (HLNPs) with poor surface activity could not adsorb at the interface to reduce IFT [20,21,22]. A great number of studies predominantly focused on the interfacial dilational rheology of silica NPs–surfactant complex [23,24,25,26]. Vatanparast et al. revealed that the non-electrostatic interactions between non-ionic surfactants and hydrophilic silica NPs exerted different and significant influences on their interfacial behaviors [27]. Eftekhari et al. found the interfacial properties of surfactant–NP mixtures were primarily determined by the surfactant–NP ratio [28].

It is worth noting that the rheological property of modified silica NPs is a particular research hotspot [29,30,31,32]. Shehab et al. employed the highly adsorbed Janus silica NPs to achieve the colloidal stability of NPs in brine and fabricate a viscoelastic gas–brine interface [33]. Zhou et al. reported adipic acid-modified silica NPs with excellent anti-temperature, anti-salinity, and interfacial activity, which dramatically reduced the oil/water IFT and increased the interfacial dilational modulus [34]. Zhang et al. investigated the interfacial behaviors of two silica NPs with different hydrophobicity. They found a nonlinear response between the IFT and the oscillating area, indicating an elastic film generated by the hydrophobic NPs at the interface [35]. Based on the above results, there should be a close relationship between the dilational rheology and the amphipathy of modified NPs. The effect of modified alkyl chain lengths on the interfacial properties of silica NPs might be responsible for the variations in their interfacial activity, which is scarcely reported.

This work synthesized a series of silica NPs with varying alkyl chain lengths modified by silane coupling agents. Various characterization methods were conducted to systematically study the properties and surface structure of MNPs. The impact of alkyl chain length on the interfacial rheological properties of these MNPs was investigated through dynamic IFT, equilibrium IFT variation, and dilational viscoelasticity experiments. Finally, the special interfacial behaviors of M6C were revealed and analyzed by the large amplitude drop compression experiment. The study has certain research significance for the relationship between the surface structure and interfacial properties of modified silica nanoparticles at the oil/water interface.

## 2. Results and Discussion

### 2.1. Characterization of MNPs

**Figure 1:**
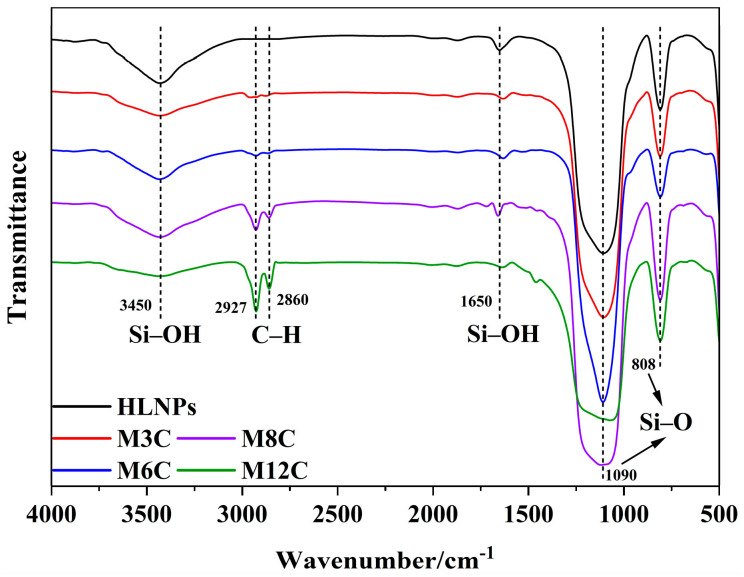
The FTIR spectra of HLNPs and various MNPs.

**Figure 2:**
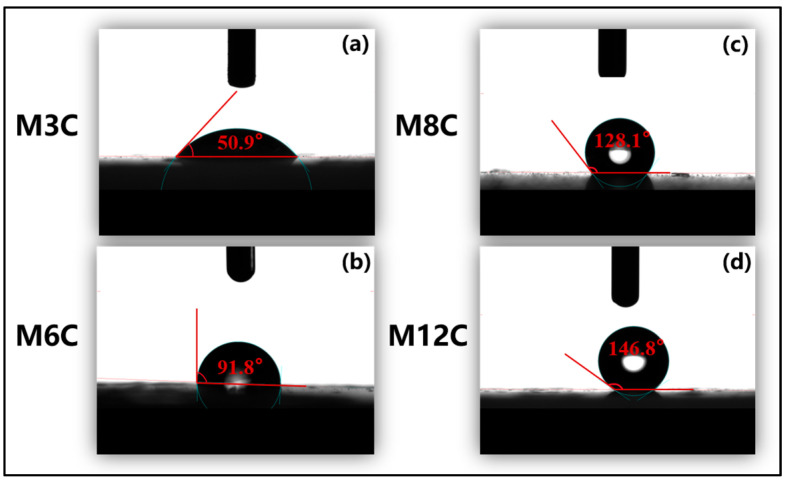
Water contact angles of different MNPs on glass at room temperature: (**a**) M3C; (**b**) M6C; (**c**) M8C; (**d**) M12C.

The FTIR spectra are employed to confirm the functional groups on the surface of the HLNPs and MNPs. (Figure 1). The stretching vibration of Si–O bonds corresponds to the double peaks at 1090 cm^−1^ and 808 cm^−1^ in all the samples [36]. The prominent and broad adsorption peaks around 3450 cm^−1^ are attributed to the Si–OH stretching vibration on the surface of the MNPs [36,37], which are evidently weakened compared to that of the HLNPs. These weak peaks at 1650 cm^−1^ are also assigned to the vibration absorption peaks of Si–OH [38]. The peaks at 2927 cm^−1^ and 2860 cm^−1^ are related to C–H vibrations of methyl and methylene, respectively [39,40]. The intensity of the peaks gradually increases with the increase in modified alkyl chain lengths, which indicates the successful fabrication of various MNPs. In addition, the water contact angles distinctly increase with the increased lengths of the modified alkyl chains (M3C (50.9°), M6C (91.8°), M8C (128.1°), and M12C (146.8°)) (Figure 2).

**Figure 3:**
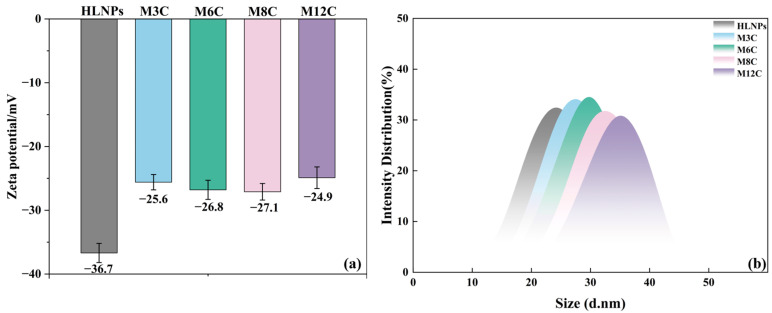
Zeta potential (**a**) values and DLS (**b**) of HLNPs and MNPs.

The Zeta potential results reflect the variation in surface groups in the HLNPs and MNPs to eliminate the effects of the modification degree of the alkyl chains (Figure 3a). The negative Zeta potential value of the HLNPs’ aqueous dispersion (−36.7 mV) is caused by the abundant hydroxyl on its surface. The grafted alkyl chain displaces part of the hydroxyl via the condensation reaction, resulting in an obvious reduction in the absolute value of the Zeta potential for all the MNP dispersions. It is worth noting that the Zeta potentials for all the MNP dispersions are similar, which indirectly confirms the similar modification degrees of the alkyl chains in the MNPs. The changed water contact angles of the MNPs can be attributed to the different lengths of their modified alkyl chains.

Figure 3b presents the dynamic light scattering (DLS) data of the HLNPs and MNPs, which can reflect the particle size distribution. It can be seen that the average particle size of the HLNPs is about 25.0 nm and the sizes of all the MNPs slightly increase, indicating the successful grafting of various alkyl chains at the MNPs surface.

Additionally, the hydroxyl density at the NPs’ surface is quantitatively measured by the titration method [41]. The consumption of NaOH solutions during the titration experiment for the HLNPs, M3C, M6C, M8C, and M12C is about 8.17 mL, 2.25 mL, 2.30 mL, 2.45 mL, and 2.19 mL, respectively. Based on formula 1, the surface hydroxyl density of the NPs can be calculated as 1.64 nm^−2^, 0.45 nm^−2^, 0.46 nm^−2^, 0.49 nm^−2^, and 0.44 nm^−2^, which should be responsible for the Zeta potential of all the MNP dispersions and directly verifies the similar modification degrees of the alkyl chains in MNPs.

### 2.2. Interfacial Behaviors of NPs at Oil/Water Interface

#### 2.2.1. Effect of NPs on Interfacial Tension

**Figure 4:**
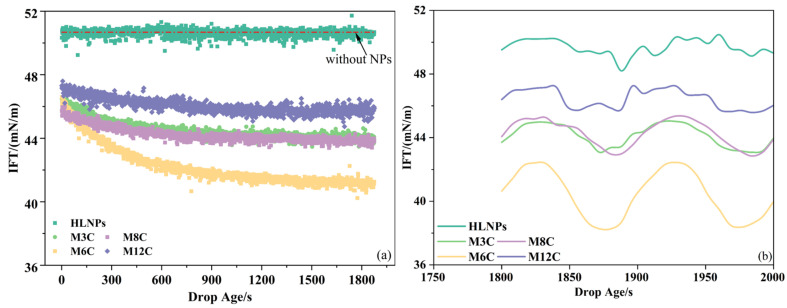
Dynamic IFTs of oil/water in the presence of HLNPs and MNPs (**a**) and their equilibrium IFT variations during sinusoidal oscillation (**b**).

**Figure A1:**
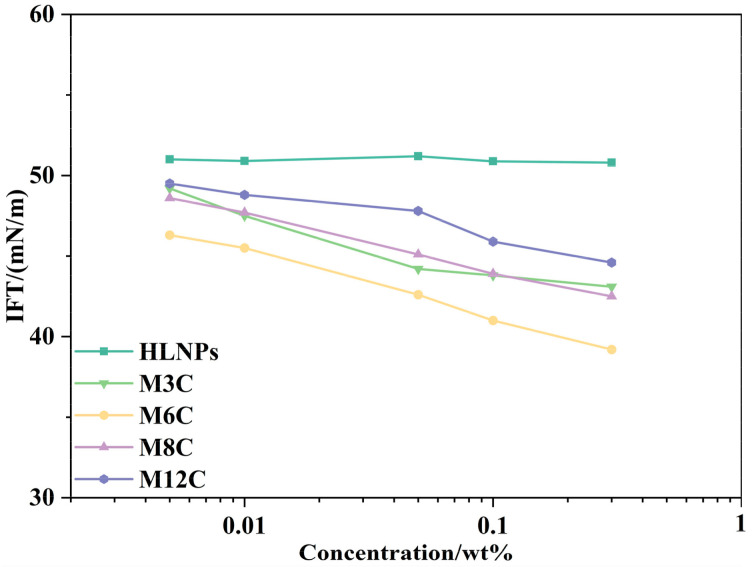
The equilibrium IFTs of HLNPs and MNPs as a function of concentration.

Figure 4 presents the oil/water IFTs in the presence of the HLNPs and MNPs. The concentrations of NPs in the bulk phase are fixed at 0.1 wt%, and the equilibrium IFTs at other concentrations are displayed in Figure A1. The oil/water ITF without NPs is about 51.0 mN/m at room temperature, which is similar to the values in some of the literature [42,43]. This is used as a control group to compare the changes in ITF caused by various NPs. The HLNPs with strong hydrophilicity mainly disperse in the aqueous phase and hardly absorb at the interface to reduce the oil/water IFT (51.0 mN/m), showing poor interfacial activity property [20,21,22].

However, the additional MNPs cause a gradual decrease in the IFT to the equilibrium value with the increase in equilibrium time, indicating the achievement of adsorption equilibrium for the MNPs at the interface within the experimental time (about 30 min). The equilibrium IFTs in the presence of M3C, M6C, M8C, and M12C are about 44.0 mN/m, 41.0 mN/m, 44.0 mN/m, and 46.0 mN/m, respectively, revealing that the equilibrium IFT first decreases and then increases with the increased length of alkyl chain grafted on the MNPs. The lowest IFT reflects the delicate hydrophilic–lipophilic balance of M6C. In addition, the water contact angle (about 90°) of the M6C further confirms its optimal interfacial activity and adsorption capacity. According to the results of the water contact angles for the MNPs, the hydrophilic–lipophilic balance of the M3C and M8C seem to be similar, showing their moderate ability to reduce the IFT (about 44.0 mN/m). Less of the M12C with excess hydrophobicity adsorbs at the interface, reflecting its poor activity and adsorption capacity.

The IFT variations in the presence of HLNPs and MNPs at low amplitude (about 10% of initial drop surface area) sinusoidal oscillation are recorded to further investigate their interfacial behaviors (Figure 4b). The magnitudes of equilibrium IFT variation in the presence of the HLNPs, M3C, M6C, M8C, and M12C are about 0.2 mN/m, 1.2 mN/m, 2.0 mN/m, 1.2 mN/m, and 0.6 mN/m, respectively. It is evident that the magnitude of equilibrium IFT variation is closely related to the interfacial activity of additional NPs. In other words, the higher interfacial activity and adsorption capacity of the MNPs retard their adsorption–desorption processes at the interface [25]. Additionally, the M6C, with the largest equilibrium IFT variation, presents the most regular response of IFT to the change in surface area compared to the other MNPs. This is because the stability of the adsorption film formed by the MNPs with excellent adsorption capacity (such as M6C) is better than those with poor adsorption capacity (such as M3C, M8C, M12C, and HLNPs) during a slight oscillation, causing the difference in sinusoidal response.

#### 2.2.2. Effect of NPs’ Concentration on Interfacial Viscoelastic Properties at Different Frequencies

**Figure 5:**
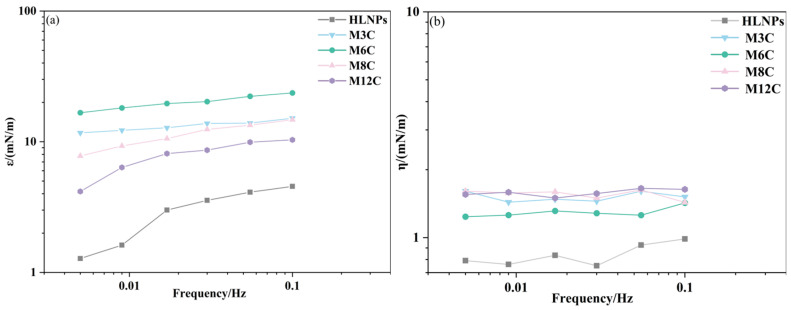
The elasticity modulus (**a**) and viscosity modulus (**b**) of NPs as a function of frequencies.

The IFT variations during drop surface sinusoidal oscillation can provide the dilational modulus of HLNPs and MNPs, revealing the interfacial rheological properties of NPs in the oil/water system. Figure 5a presents the change in elasticity modulus for NPs as a function of oscillating frequencies, which is a significant parameter in affecting the interfacial rheology. It is demonstrated that the elasticity modulus of NP film depends on frequency, and the values gradually augment with the increase in frequency. The increasing elasticity modulus indicates that the diffusion exchange process of particles plays a crucial role at lower bulk concentrations [35]. As shown in Figure 5b, there seems to be no evident relationship between the viscosity modulus of MNPs and the oscillation frequency of the drop. The MNPs with interfacial activity can absorb at the interface and form an adsorption film, and the viscoelasticity of the film is determined by both elastic modulus and viscous modulus. The results in Figure 5 show that the elasticity modulus of the MNPs is significantly higher than their viscosity modulus, which indicates that the adsorption film formed by the MNPs at the interface is dominated by elasticity.

Compared to conventional surfactants, NPs can accumulate at the drop bottom to cause irregular dilational deformation of the drop surface and inaccurate IFT variations, especially at high concentrations [44]. The interfacial rheological properties of NPs are dramatically affected by their concentrations, so it is advisable to keep the concentration of the system below 0.5 wt% to avoid a severe accumulation of NPs.

**Figure 6:**
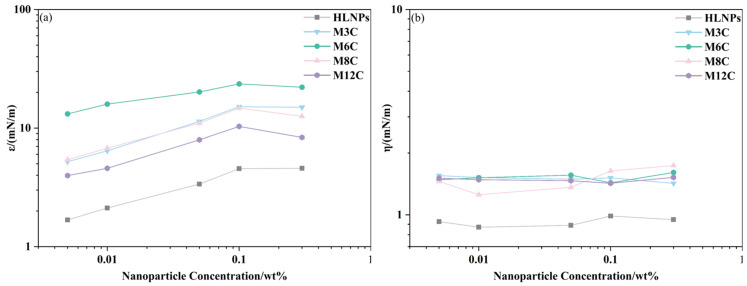
The elasticity modulus (**a**) and viscosity modulus (**b**) of NPs as a function of concentration.

**Figure A2:**
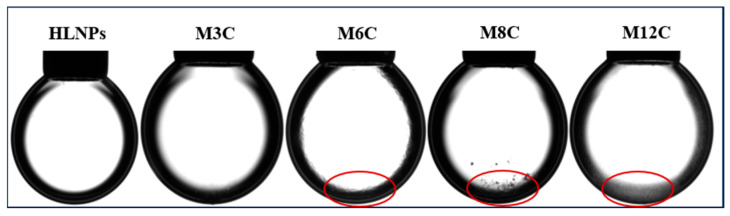
Morphologies of different NPs droplets with a 12 μL concentration, 0.3 wt%, after standing 30 min. (The red circles represent the obvious deposition of NPs).

The dilational elasticity modulus and viscosity modulus of NPs are studied as a function of concentration, and the results are plotted in Figure 6. Different from the changes caused by frequency variation, the elasticity modulus of the NPs first increases and then decreases with increasing concentration. The effects of concentration on the elasticity modulus are more complicated. The NPs adsorbed at the interface gradually increase at higher concentrations and form a two-dimensional network structure [44,45] at concentrations below 0.1 wt%, which contributes to the increase in elasticity modulus, while the elasticity modulus of NPs remains unchanged or decreases when the concentration increases to 0.3 wt%. This may be attributed to the change in drop shape and the destruction of the network structure at the interface caused by the aggregation of NPs at the higher concentration, resulting in the decline in modulus (Figure A2). Thus, the elasticity modulus of NPs would pass through the maximum value at a critical concentration (between 0.1 wt% and 0.3 wt%), which is similar to the results of Zhang [35]. In addition, the viscosity modulus remains at a much lower level than the elasticity modulus in the experimental concentration range, which also indicates that the adsorption film formed by the MNPs at the interface is dominated by elasticity.

#### 2.2.3. Effect of Alkyl Chain Length on Dilational Elasticity for MNPs

**Figure 7:**
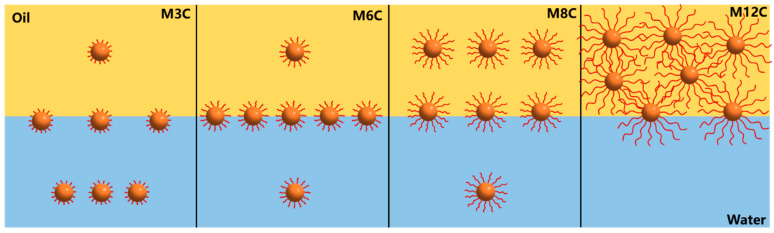
The adsorption models of MNPs with diverse alkyl chains at the oil/water interface.

Based on the above results, the dilational elasticity modulus of the MNPs first increases and then decreases with the increased length of the alkyl chain (Figure 5a and Figure 6a). Previous studies demonstrated that the improved hydrophobicity could strengthen the interfacial interaction and activity of NPs, leading to an increase in elasticity modulus. In the present system, the longer modified alkyl chains cause the spontaneous movements of MNPs with excessive hydrophobicity (M8C, M12C) into the oil phase, which decreases the activity and elasticity modulus. Figure 7 presents the adsorption models of various MNPs at the oil/water interface. The M6 with the optimal interfacial activity possesses the largest elasticity modulus, and the modulus value of the M3C with the shorter alkyl chain approaches that of the M8C with the longer chain due to the similar interface activity. Meanwhile, the excessively hydrophobic M12C mainly distributes in the oil phase, and the low elasticity modulus should be attributed to less adsorbed M12C caused by the Brownian motion.

### 2.3. Special Interfacial Behaviors of M6C in Large Amplitude Compression Experiment

**Figure 8:**
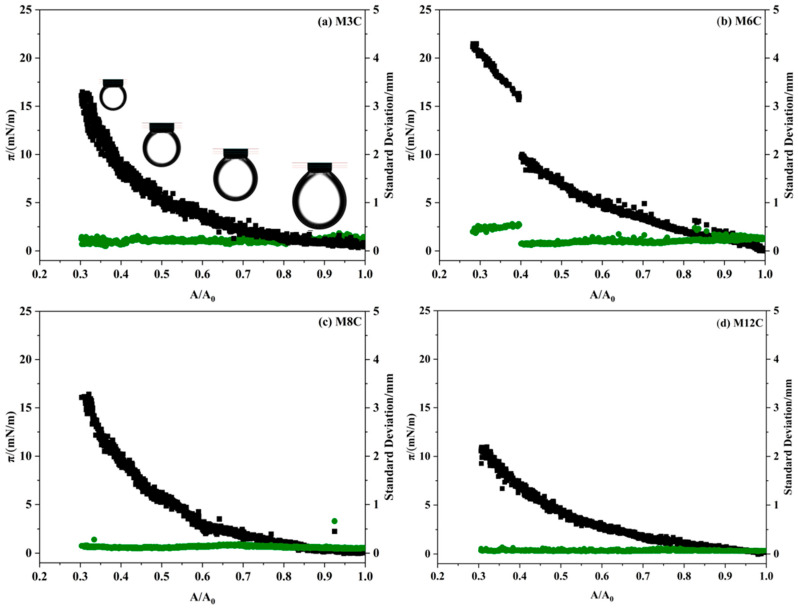
Surface pressure (black line) and standard deviation (green line) changes during drop compression for the MNPs of varying hydrophobicity (0.1 wt%).

The drop compression experiment is conducted after the IFT reaches the equilibrium value to reflect the adsorption–desorption processes of the MNPs at the interface [16,25,46]. The variations in surface pressure and standard deviation for MNPs in response to the variations in the interfacial area are depicted in Figure 8. The horizontal axis represents the normalized surface area of the drop with respect to the initial value. It is found that the surface pressure values of all the systems significantly increase with the compression process of the drop, indicating the increased amount of MNPs adsorbed at the interface. The surface pressure values are closely related to the modified alkyl chain lengths of the MNPs, and the final surface pressure of M3C, M6C, M8C, and M12C are about 16.5 mN/m, 21.2 mN/m, 16.4 mN/m, and 10.9 mN/m, respectively. The greatest interfacial activity and adsorption capacity of M6C should be responsible for its maximum surface pressure, which is consistent with the proposed adsorption models in Figure 7.

Notably, the surface pressure of the M6C discontinuously and sharply increases at the surface ratio of 0.4, whereas the pressure curves continuously and gradually increase for the other MNPs. During the drop compression process, some adsorbed MNPs stay at the oil/water interface, and others can move to the bulk phase, which depends on the hydrophilic–lipophilic balance of the MNPs. For the more hydrophilic M3C and more hydrophobic M8/M12, the electrostatic repulsion among NPs causes their gradual desorption from the interface with the decrease in drop surface area, resulting in the generation of continuous pressure curves, while the M6C can generate a more close-packed adsorption layer at the initial compression process of the drop (the surface ratio > 0.4), which is reflected by the larger increase in the surface pressure. When the surface ratio is about 0.4, the adsorbed M6C with the optimal amphipathy can partly overcome the electrostatic repulsion among them, resulting in the more close-packed arrangements of the M6C and the dramatic change in surface pressure. In addition, the standard deviation of fitting the Laplace equation to the experimental drop profile is dependent on the change in drop shape during the compression process. The standard deviations in the M3C, M8C, and M12C systems are much lower (<0.3 mm), indicating the gradual compression of the adsorbed NPs at the interface. However, the discontinuity in the M6C standard deviation should be attributed to the more close-packed rearrangements of NPs at the drop surface.

**Figure 9:**
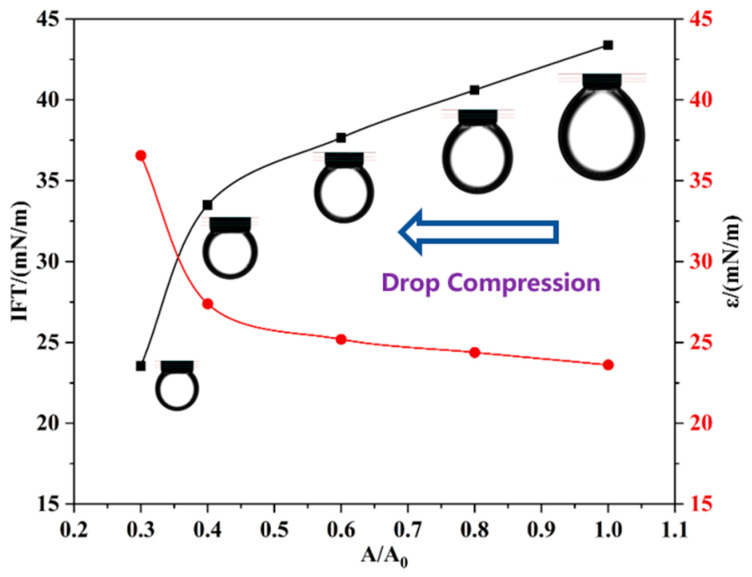
The variations in IFT and dilational elasticity during the large compression path of M6C.

To confirm the proposed mechanism, the variations in IFT and dilational elasticity are measured during the compression process (Figure 9). The compression process of the drop increases the amount of M6C adsorbed at the interface, which leads to a linear decrease in IFT and an increase in the dilational elasticity modulus before the surface ratio drops to 0.4. After the critical point, the dramatic variations in IFT and the elasticity modulus are attributed to the sharply increased amount of adsorbed M6C that overcomes the electrostatic repulsion, which further verifies the special interfacial behaviors of the M6C during the large amplitude compression experiment.

## 3. Experimental Section

### 3.1. Materials

Nano-fumed silica (99.8%, 150 m^2^/g, 7~40 nm), trimethoxy(propyl)silane (98%), trimethoxy(hexyl)silane (98%), trimethoxy(octyl)silane (97%), trimethoxy(dodecyl)silane (93%), and n-octane (96%) were purchased from Shanghai Aladdin Biochemical Technology Co., Ltd. (Shanghai, China). Sodium chloride (NaCl, 99.5%), sodium hydroxide (NaOH, 97%), hydrochloric acid (HCl, 36%~38%), and anhydrous ethanol (99.7%) were bought from Sinopharm Chemical Reagent Co., Ltd. (Shanghai, China). All the chemical reagents were employed directly without further purification. Deionized (DI) water was used in all the experiments. The HLNPs, M3C, and M6C were dispersed in water, and the M8C and M12C with high alkyl chain lengths were dispersed in n-octane, before being sonicated for 30 min before each measurement to break down any aggregation and sedimentation. The abbreviations for all the nanoparticles are shown in Table A1.

### 3.2. Synthesis of MNPs with Various Alkyl Chain Lengths

A series of MNPs were prepared via the modification of HLNPs by different silane coupling agents [47,48,49]. First, about 0.5 g of HLNPs, 1.0 g of trimethoxy(propyl)silane, and 2.0 g of DI water were added into 30 mL of anhydrous ethanol in a round-bottomed flask. Then, the pH of the mixture was adjusted to 2~3 with 0.1 mol/L of HCl solution, and the mixture was heated to 70 °C for 3 h with reflux and agitation. After the reaction, the mixture was washed and centrifuged with anhydrous ethanol to remove the impurities. Finally, the product was dried at 75 °C for 24 h to acquire MNPs with propyl alkyl chains (M3C). Likewise, the M6C, M8C, and M12C were prepared with trimethoxy(hexyl)silane, trimethoxy(octyl)silane, and trimethoxy(dodecyl)silane, respectively.

### 3.3. Characterization of MNPs

The Fourier transform infrared spectra (FTIR) were employed to reflect the functional groups of NPs in the 500–4000 cm^−1^ wavelength range with the PerkinElmer Spectrum Two spectrometer (PerkinElmer, Waltham, MA, USA). The optical contact angle measuring and contour analysis systems OCA15EC (Dataphysics, Filderstadt, Germany) were utilized to measure the water contact angles of MNPs deposited on glass. The Zeta potential of the HLNPs and MNPs was measured to reveal their variation in surface groups by dynamic light scattering (Malvern, UK). The sizes of the NPs were determined by a laser particle size analyzer (Brookhaven, NanoBrook Omni, Holtsville, NY, USA).

The hydroxyl density at the NPs’ surface was measured by a titration experiment. Approximately 2.0 g of silica NPs, 75.0 g of 20% NaCl solution, and 25 mL of anhydrous ethanol were added to a 200 mL beaker, and the pH of the mixture was adjusted to 4 with 0.1 mol/L HCl or NaOH solution. Then, a 0.1 mol/L NaOH solution was used to adjust the pH of the mixture to 9, and its volume was recorded. The number of hydroxyl groups on the particle surface can be calculated by Equation (1) [41]:

(1) $$N=\frac{CVN_{A}\times 10^{-3}}{Sm}$$

*N* is the surface hydroxyl number, *C* is the NaOH concentration (0.1 mol/L), *V* is the consumed NaOH volume (mL) when the pH increases from 4.0 to 9.0, *N_A_* is the Avogadro’s number, *S* is the surface area of the sample (nm^2^/g), and m is the quantity of silica NPs (g).

### 3.4. Interfacial Tension and Dilational Rheology Experiments

IFT and dilational rheology tests were performed to investigate the interfacial behaviors of NPs using the OCA15EC (Dataphysics, Germany) [50,51]. The basic operation and measurement principles of the experiments were as follows. First, a 12 μL volume drop of the solution was prepared through a microsyringe with a 1.5 mm diameter stainless steel needle and held vertically in a quartz colorimetric dish filled with the oil phase. Then, the profile of the drop was captured by a charge-coupled device (CCD) camera, and the image of the drop was analyzed using the Laplace equation to obtain the IFT data. The IFT of the HNPs and MNPs was recorded for 30 min until reaching equilibrium. In order to study the dilational rheological properties of the interface, the dilational viscoelastic modulus of the NP-stabilized interface was obtained by analyzing the equilibrium IFT variation caused by a slight perturbation in the formed drop. Based on this principle, the variation in the viscoelastic modulus, including frequency and concentration, was studied using a sinusoidal oscillation with an amplitude of 10% of the initial drop surface area. In addition, the adsorption process of MNPs and interaction at the interface was investigated by a large linear compression (about 70% of the initial drop surface area) experiment at the rate of 0.01 μL/s. All the experiments were conducted at room temperature.

## 4. Conclusions

In conclusion, we successfully synthesized a series of MNPs with different alkyl chain lengths and studied their interfacial behaviors via the interfacial dilational rheological experiments. The M6C with a moderate alkyl chain length exhibited the optimal interfacial activity and adsorption capacity to reduce the IFT to 41.0 mN/m, which was verified by the equilibrium IFT variations in the MNPs and the irregular response of IFT to the change in surface area. Additionally, the frequency-dependence of dilational rheological elasticity was attributed to the diffusion exchange of the NPs, while the continuous increase in the MNPs’ elasticity modulus in this system indicated the interaction among NPs was dominant compared with the diffusion exchange of NPs at lower concentrations. The nonlinear variation in elasticity modulus with an increasing alkyl chain revealed the structure and amphipathy of NPs were important factors in generating highly elastic films. Finally, the large amplitude compression experiment reflected the M6C with the optimal amphipathy could partly overcome the electrostatic repulsion among close-packed particles and cause the sharp increase in adsorbed NPs at the surface, leading to the special interfacial behaviors of the discontinuous surface pressure and standard deviation. This study shows a simple and promising approach to constructing MNPs with various alkyl chains, which is of great significance in investigating the relationship between their interfacial rheological properties and structures.

## Data Availability

The data presented in this study are available on request from the corresponding authors.

## Appendix A

**Table A1:** Nomenclature used in this article.

Acronyms	
NPs	Nanoparticles
HLNPs	Hydrophilic silica nanoparticles
MNPs	Modified silica nanoparticles
M3C	MNPs with propyl alkyl chain
M6C	MNPs with hexyl alkyl chain
M8C	MNPs with octyl alkyl chain
M12C	MNPs with dodecyl alkyl chain

Figure A1 presents the equilibrium IFTs of HLNPs and MNPs in a range of concentrations, and the measurement of each data point was conducted after the droplet stood for 30 min. It is obvious that the equilibrium IFTs of NPs decrease with the increased concentration. Some MNPs form aggregates and accumulate at the bottom of the droplet when the bulk concentration reaches 0.3 wt%, which can be seen in Figure A2. In the present study, 0.1 wt% is the concentration where the IFT of NPs decreases the most without visible aggregation, so this concentration is used in the dynamic interfacial tension and rheological measurements in this paper.
